# Common variants of a urate-associated gene *LRP2* are not associated with gout susceptibility

**DOI:** 10.1007/s00296-013-2924-8

**Published:** 2013-12-24

**Authors:** Akiyoshi Nakayama, Hirotaka Matsuo, Toru Shimizu, Yuzo Takada, Takahiro Nakamura, Seiko Shimizu, Toshinori Chiba, Masayuki Sakiyama, Mariko Naito, Emi Morita, Kimiyoshi Ichida, Nariyoshi Shinomiya

**Affiliations:** 1 Department of Integrative Physiology and Bio-Nano Medicine, National Defense Medical College, 3-2 Namiki, Tokorozawa, Saitama 359-8513 Japan; 2 Medical Group, Headquarters, Iwo-to Air Base Group, Japan Air Self-Defense Force, Ogasawara, Japan; 3 Midorigaoka Hospital, Takatsuki, Japan; 4 Laboratory for Biofunctions, The Central Research Institute, National Defense Medical College, Tokorozawa, Japan; 5 Laboratory for Mathematics, National Defense Medical College, Tokorozawa, Japan; 6 Department of Preventive Medicine, Graduate School of Medicine, Nagoya University, Nagoya, Japan; 7 Department of Internal Medicine, Jikei University School of Medicine, Tokyo, Japan; 8 Department of Pathophysiology, Tokyo University of Pharmacy and Life Sciences, Tokyo, Japan

**Keywords:** Gouty arthritis, Hyperuricemia, Hyperlipidemia, Urate exporter, Low-density lipoprotein receptor (LDLR), LDLR gene family

## Abstract

A recent genome-wide association study revealed that there is an association between serum uric acid (SUA) levels and rs2544390, a common variant in low-density lipoprotein-related protein 2 (*LRP2*/*Megalin*) gene. Two other variants of *LRP2*, rs2229268 and rs3755166, are also found to have associations with dyslipidemia and Alzheimer’s disease, respectively, which also could have a relationship with SUA in human. Although no studies report that LRP2 transports urate, LRP2 is a multi-ligand receptor and expresses in many tissues including kidney, suggesting a direct and/or indirect relationship with gout. In the present study, we investigated the association between gout and these variants of *LRP2* with 741 clinically diagnosed male gout patients and 1,302 controls. As a result, the three common *LRP2* variants, rs2544390, rs2229268 and rs3755166, showed no association with gout (*P* = 0.76, 0.55, and 0.22, respectively). Our study is the first to reveal that an SUA-related gene *LRP2* is not involved in gout susceptibility.

## Introduction

Gout is a common disease as a consequence of hyperuricemia. A recent genome-wide association study (GWAS) [1] with 8,868 Japanese revealed the association between serum uric acid (SUA) levels and rs2544390, which is a single nucleotide polymorphism (SNP) in low-density lipoprotein-related protein 2 (*LRP2*, also known as *Megalin*). *LRP2* is a member of the low-density lipoprotein receptor [*LDLR* (MIM606945)] gene family, and two SNPs of *LRP2*, rs2229268 and rs3755166, are also found to have associations with dyslipidemia [2] and Alzheimer’s disease [3], respectively. In this study, we investigated the association between gout and these SNPs with clinically diagnosed gout patients and controls.

## Subjects and methods

### Subjects

All procedures were carried out in accordance with the standards of the institutional ethical committees involved in this project and the Declaration of Helsinki. Written informed consent was obtained from each subject participating in this study. As gout cases, 741 Japanese male individuals were collected from the outpatients of the gout clinics in either Jikei University Hospital (Tokyo, Japan) or Midorigaoka Hospital (Osaka, Japan). All of them were clinically diagnosed with primary gout according to the criteria established by the American College of Rheumatology [4]. As a control group, 1,302 Japanese male individuals with normal SUA (≤7.0 mg/dl) without gout history were collected from the Japan Multi-Institutional Collaborative Cohort Study (J-MICC Study) [5]. The mean age and body-mass index with standard deviation are 55.0 ± 13.2 years old and 24.6 ± 3.5 kg/m^2^ for cases, respectively, and 52.7 ± 8.4 years old and 23.2 ± 2.8 kg/m^2^ for controls, respectively.

### Genetic analysis and statistical analysis

Genomic DNA was extracted from whole peripheral blood cells [6]. Genotyping of rs2544390, rs2229268, and rs3755166 in *LRP2* gene was performed by an allelic discrimination assay (Custom Taqman MGB, Applied Biosystems) with a LightCycler 480 (Roche Diagnostics) [7]. To confirm their genotypes, more than 25 samples were subjected to direct sequencing with the following primers: for rs2544390, forward 5′-CTGTCTGAGACCATGACACAG-3′ and reverse, 5′-CCTCACCTGTCATTGTCTTG-3′; for 2229268, forward 5′-TCCGTTCAACTTTCAGACAG-3′ and reverse 5′-TTCCCAACTTTCTCAGGTTAC-3′; for 3755166, forward 5′-GTGTAAGGCCACTTGTGC-3′ and reverse 5′-GAAATGGACGAGGAGGAAAG-3′. DNA sequencing analysis was performed with a 3130xl Genetic Analyzer (Applied Biosystems) [8].

The software R (version 3.0.2) (http://www.r-project.org/) with package GenABEL was used for the calculation of linkage disequilibrium (*r* ^2^). For the calculations in the statistical analyses, SPSS v.17.0J (IBM Japan Inc., Tokyo, Japan) was used. The χ^2^ test was used for association analysis.

## Results

Table 1 shows the alleles of *LRP2* variants, rs2544390, rs2229268, and rs3755166, for 2,043 Japanese participants (741 gout cases and 1,302 controls). The call rates for rs2544390, rs3755166, and rs2229268 were 98.2, 98.1, and 99.5 %, respectively. *P* values for Hardy–Weinberg equilibrium of these SNPs were 0.039, 0.095, and 0.134. *P* values that suggested mistyping were not obtained. The minor allele frequencies (MAFs) for the three *LRP2* variants were more than 0.27 in both case and control groups, indicating these SNPs are very common in both these groups. No strong linkage disequilibrium was observed between these three SNPs (*r* ^2^ = 0.0014 between rs2544390 and rs2229268, *r* ^2^ = 0.0013 between rs2229268 and rs3755166, *r* ^2^ = 0.15 between rs3755166 and rs2544390, respectively), showing that these SNPs are independent of each other.

**Table 1:** Association analysis of *LRP2* variants, rs2544390, rs2229268, and rs3755166 in gout patients

	Chromosomal positions^a^ (bp)	Alleles^b^						*P* value	OR	95 % CI
		Case			Control					
		1	2	MAF	1	2	MAF			
rs2544390	170204846	747	717	0.490	1,314	1,236	0.485	0.758	1.020	0.897–1.160
rs2229268	170025083	1,051	423	0.287	1,829	705	0.278	0.552	1.044	0.906–1.204
rs3755166	170219881	702	778	0.474	1,277	1,307	0.494	0.223	1.083	0.953–1.231

*MAF* minor allele frequency, *OR* odds ratio, *CI* confidence interval

^a^SNP positions are based on NCBI human genome reference sequence build 37.5. *LRP2* is located on chromosome 2q31.1

^b^The major allele was referred to as allele 1 and the minor allele as 2. Allele 1 is C and allele 2 is T in rs2544390. Allele 1 is A and allele 2 is G in rs2229268. Allele 1 is A and allele 2 is G in rs3755166

For all gout cases, the association analyses of the three *LRP2* variants, rs2544390, rs2229268, and rs3755166, showed no association with gout (Table 1).

## Discussion

Our study demonstrated that the three *LRP2* variants, rs2544390, rs2229268, and rs3755166, had no association with gout.

Recent GWAS of SUA [9, 10] identified several genes including *GLUT9*/*SLC2A9* and *ABCG2*/*BCRP*, which have been revealed to have associations with urate disorders such as renal hypouricemia [11, 12] and gout [13, 14]. Recent reports also show the significance of transporter genes such as *ABCG2* [15, 16], *NPT1*/*SLC17A1* [17], *MCT9*/*SLC16A9* [18], and *OAT4*/*SLC22A11* [19], for the pathogenesis of gout. *LRP2* was first reported to have the association with SUA in the GWAS by Kamatani et al. [1]. Although we found no studies reporting that LRP2 transports urate, *LRP2* variants could have an association with gout risks because gout is a consequence of hyperuricemia. Moreover, it is also demonstrated that LRP2 is a multi-ligand receptor and is expressed in various tissues, mainly in the kidney, especially in glomeruli and proximal tubular cells. As LRP2 has a role of renal reabsorption for its ligands such as insulin [20], *LRP2* variants could have an association with SUA variation with increasing insulin resistance.

LRP2 is originally found as a member of the low-density lipoprotein (LDL) receptor family and has been suggested to mediate endocytosis of LDL. Indeed, Mii et al. [2] reported that one variant of *LRP2*, rs2229268, has an association with serum LDL levels in humans, indicating the direct association between *LRP2* and LDL. Since dyslipidemia is known as a risk to increase the insulin resistance, rs2229268 seems to have an association with SUA variation. Otherwise, *LRP2* could be associated with SUA variation through the endocytosis of urate-binding proteins.

Interestingly, Wang et al. [3] previously reported the association between rs3755166 in *LRP2* and Alzheimer’s disease in a Chinese population. LRP2, whose ligand ApoE [21] is known for the risk of Alzheimer’s disease [22, 23], is expressed in brain and facilitates the clearance of the Aβ peptide, that is, the cause of Alzheimer’s disease [24]. Together with the fact that urate has anti-oxidant effects, *LRP2* variants carrying the risk of Alzheimer’s disease might have an association with SUA variation.

However, the present study first revealed that the common variants of *LRP2* have no association with gout susceptibility. Although *LRP2* was first reported to have an association with SUA in Japanese population [1], there are no replication studies indicating an association between *LRP2* and SUA in other ancestry such as a European population. It is possible that the present study failed to show these associations due to the limited sample (2,043 individuals). Although further studies of *LRP2* are necessary to reveal the relationship between *LRP2* variants and gout, our study at least revealed that *LRP2* is not a strong genetic risk for gout.
